# Superwicking Functionality of Femtosecond Laser Textured Aluminum at High Temperatures

**DOI:** 10.3390/nano11112964

**Published:** 2021-11-04

**Authors:** Ranran Fang, Xianhang Zhang, Jiangen Zheng, Zhonglin Pan, Chen Yang, Lianrui Deng, Rui Li, Chunhong Lai, Wensheng Yan, Valeriy S. Maisotsenko, Anatoliy Y. Vorobyev

**Affiliations:** 1 School of Optoelectronic Engineering, Chongqing University of Posts and Telecommunications, 2 Chongwen Road, Chongqing 400065, China; fangrr@cqupt.edu.cn (R.F.); s190402010@stu.cqupt.edu.cn (X.Z.); zhengjg@cqupt.edu.cn (J.Z.); laich@cqupt.edu.cn (C.L.); yws118@gmail.com (W.Y.); 2 School of Science, Chongqing University of Posts and Telecommunications, 2 Chongwen Road, Chongqing 400065, China; pzl15185309575@163.com (Z.P.); y2379202627@163.com (C.Y.); DLR20010527@163.com (L.D.); 3 School of Automation, Chongqing University of Posts and Telecommunications, 2 Chongwen Road, Chongqing 400065, China; lirui@cqupt.edu.cn; 4 M-Cycle Corporation, 1120 Delaware St. #110, Denver, CO 80204, USA; valeriymaisotsenko@gmail.com

**Keywords:** wicking materials, aluminum, femtosecond laser processing, nanostructures, microstructures, laser-induced periodic surface structures (LIPSS), capillarity flow dynamics, cooling of electronics, Maisotsenko cycle, global climate change

## Abstract

An advanced superwicking aluminum material based on a microgroove surface structure textured with both laser-induced periodic surface structures and fine microholes was produced by direct femtosecond laser nano/microstructuring technology. The created material demonstrates excellent wicking performance in a temperature range of 23 to 120 °C. The experiments on wicking dynamics show a record-high velocity of water spreading that achieves about 450 mm/s at 23 °C and 320 mm/s at 120 °C when the spreading water undergoes intensive boiling. The lifetime of classic Washburn capillary flow dynamics shortens as the temperature increases up to 80 °C. The effects of evaporation and boiling on water spreading become significant above 80 °C, resulting in vanishing of Washburn’s dynamics. Both the inertial and visco-inertial flow regimes are insignificantly affected by evaporation at temperatures below the boiling point of water. The boiling effect on the inertial regime is small at 120 °C; however, its effect on the visco-inertial regime is essential. The created material with effective wicking performance under water boiling conditions can find applications in Maisotsenko cycle (M-cycle) high-temperature heat/mass exchangers for enhancing power generation efficiency that is an important factor in reducing CO_2_ emissions and mitigation of the global climate change.

## 1. Introduction

Commonly, the direct laser ablation method produces wicking materials with hierarchical capillary structures composed of a basic microstructure (typically, microgrooves or micropillars) and a random fine micro/nanotexture on the surface of the basic microstructure [1]. The hierarchical property of the laser-produced capillary structures comes from the nature of direct femtosecond laser ablation that intrinsically induces random surface structures on nano- and micro-scales [2,3,4,5,6,7,8,9,10]. Previous studies have been mainly focused on the optimization of the basic capillary microstructures with random nano/microtextures [1,11,12,13,14,15,16,17,18]. In addition to the random nano/microtextures, the direct laser ablation also allows producing regular surface nano/microstructures (referred to as laser-induced periodic surface structures (LIPSS) or ripples) through surface plasmon polariton or some sort of self-organization mechanisms [1,19,20,21,22,23,24,25,26,27,28,29,30,31,32,33,34,35,36,37,38,39,40,41]. LIPSSs have found many applications in the modification of the optical [42,43,44,45,46,47,48,49,50,51,52], wetting [53,54], biomedical [55,56,57,58], catalytic [59], tribological [60], superconducting [61], and other [62,63] properties of materials. Recently, it has been demonstrated that LIPSSs produced on the surface of micropillars and microholes enhance the wicking functionality of a micropillar/microhole array structure fabricated by direct femtosecond laser writing on a Ti-6Al-4V alloy surface [64]. Here, we apply the LIPSS texturing approach to enhance the capillary action of an array of parallel microgrooves on aluminum. In addition to LIPSSs, we also produce random fine microholes on the ridges and valleys of the microgrooves for improving the stability of the capillary performance at high temperatures. These fine microholes play the role of microreservoirs, supplying water to areas with intensive evaporation and preventing dry-out spot formation in cooling applications. Our research on efficient wicking materials is motivated by a large variety of their applications in such areas as the thermal management of high-heat flux semiconductor electronics [65], cooling data centers [66,67], energy-harvesting [68], thermal management of robots [69], water desalination [18,70], waste heat recovery [71,72,73], spacecraft thermal management [74,75], and Maisotsenko cycle (M-cycle) technologies [76,77,78,79]. Our choice of aluminum is stimulated by its long-term stable wicking properties due to the formation of a hydrophilic aluminum oxide hydroxide [γ–AlO(OH)] surface layer (referred to as the Boehmite layer) caused by the chemical interaction of aluminum with hot water that improves both the hydrophilic and corrosion-resistance properties of Al wicks [80,81,82,83]. Previously, capillary flow dynamics in microgrooves produced on an aluminum surface by femtosecond laser pulses has been studied at the stage of the classic Washburn flow at room temperature [15,16,18]. However, many important applications require materials with efficient wicking functionality at high temperatures. In this paper, we study the wicking performance of our material in a temperature range between 23 and 120 °C. Furthermore, our study of the capillary flow dynamics includes the inertial and visco-inertial stages occurring prior to Washburn’s regime of liquid spreading. Understanding the capillary flow in the initial stages is important for preventing dry-out spots in cooling high-heat flux electronic devices of 4G/5G telecom networks [65] and in designing miniature lab-on-chips, where the length of the capillary channel is so small that the Washburn stage does not take place [84].

The results of our study on capillary water spreading dynamics show that the created array of microgrooves with both LIPSS and fine microhole textures provides a record-high velocity of water transport, reaching about 450–500 mm/s in the inertial stage of capillary flow; for a comparison, the highest water spreading velocity of 370 mm/s in capillary microgrooves has been previously reported in [85]. Even at 120 °C, when the spreading water intensively boils, the maximum capillary flow velocity remains extraordinarily high, achieving about 320 mm/s. The developed hierarchical wicking material outperforms the other reported materials in terms of the wicking performance in a wide temperature range, indicating its practical potential for a broad range of applications mentioned above. An important issue is that applications of the created material in M-cycle technologies for air-conditioning and power generation sectors can essentially increase energy efficiency in these sectors, thus reducing greenhouse gas emissions and contributing to the mitigation of the global climate change.

## 2. Experimental Section: Fabrication and Characterization

The capillary 1D array of parallel microgrooves on the surface of aluminum is produced using a femtosecond laser system (Astrella, Coherent Inc., Santa Clara, CA, USA) that generates 86 fs pulses with energy of 7.13 mJ/pulse at a maximum repetition rate of 1 kHz with a central wavelength of 800 nm. The laser beam is focused with an achromatic lens onto an aluminum sample mounted on a computer-controlled XYZ translation stage. A half-wave plate and polarizing beam-splitter cube are used for varying the laser power. The 1D array of parallel microgrooves is produced by raster scanning of the sample across the laser beam. To find efficient wicking surface structures, we vary laser fluence *F*, step between scanning lines, scanning speed, focal spot diameter, and pulse repetition rate. In the result of these tests, we find that the most efficient wicking performance is observed for microgrooves containing LIPSS-textured areas and microholes on both valleys and ridges of the microgrooves. In our study, this structure is produced using laser fluence of 8.6 J/cm^2^, step between scanning lines of 100 µm, pulse repetition rate of 1000 Hz, and scanning speed of 1 mm/s. Aluminum plates purchased from Goodfellow are processed in the ambient air at temperature of 23 °C and relative humidity of 50% measured using a humidity sensor HMP7 from Vaisala (Helsinki, Finland). The dimensions of the laser-textured surface area are 20 × 50 mm. After laser processing, the samples were cleaned with alcohol and deionized water in an ultrasonic bath for 3 min in each liquid for removing redeposited debris and dried by blowing compressed nitrogen. The structural features of the produced wicking structure are studied using a 3D laser scanning microscope VK-X1100 from Keyence and scanning electron microscope (SEM) Sigma 300 from Zeiss (Jena, Germany). Elemental composition of both treated and untreated sample surfaces is examined by an energy dispersive X-ray spectroscopy (EDS) using a Brucker XFlash 6/30 detector (Karlsruhe, Germany). In our study, we prepare three Al samples (#1, #2, and #3) using the same laser processing parameters. Sample #1 is studied as after laser processing. Samples #2 and #3 are further treated in hot water at 82 and 100 °C for 15 min, respectively, to form an AlO(OH) surface layer. These hot water treatments are similar to those used in [80].

**Figure 1:**
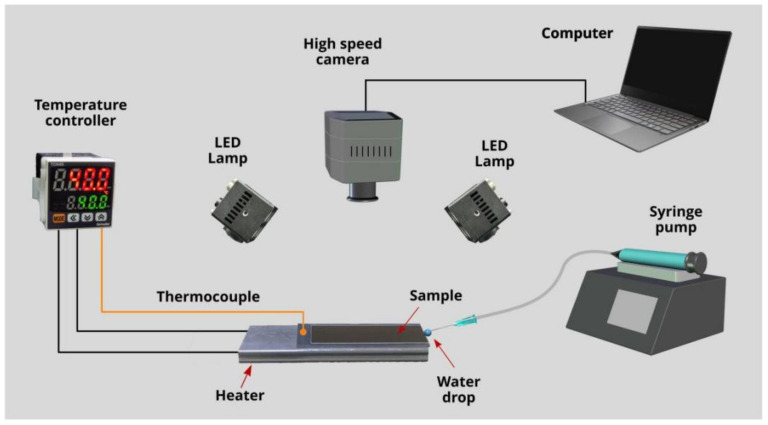
Experimental setup for high-speed video imaging of water spreading on a wicking surface at various temperatures.

The wicking functionality of the produced samples is studied by video imaging capillary spreading of deionized water on a horizontally positioned sample. The video imaging setup is shown in Figure 1. An aluminum sample is mounted on a heater. The temperature of the sample surface is regulated by a temperature controller (TCN4S from Autonics, Busan, South Korea) and thermocouple (5TC-TT-K-30-36 from Omega, Norwalk, CT, USA) attached onto untreated sample surface. Water is supplied to the wicking surface structure from a pendant 10-µL drop formed by a syringe pump Elite 11 from Harvard Apparatus Inc. (Holliston, MA, USA). The capillary flow of water is captured by a high-speed VEO 710L Phantom camera at a rate of 1000 frames per second (fps). The wicking dynamics are studied at sample temperatures *T* = 23, 40, 60, 80, 100, and 120 °C under ambient air conditions with relative humidity of 50% and temperature of 23 °C. Using video recordings of water flow, we find both water spreading distance *h* and water spreading velocity as a function of time *t*, which we obtain as a numerical derivative Δ*h*/Δ*t*, where Δ*h* is the difference of spreading distance between two consecutive video frames and Δ*t* = 10^−3^ s.

## 3. Results and Discussion

**Figure 2:**
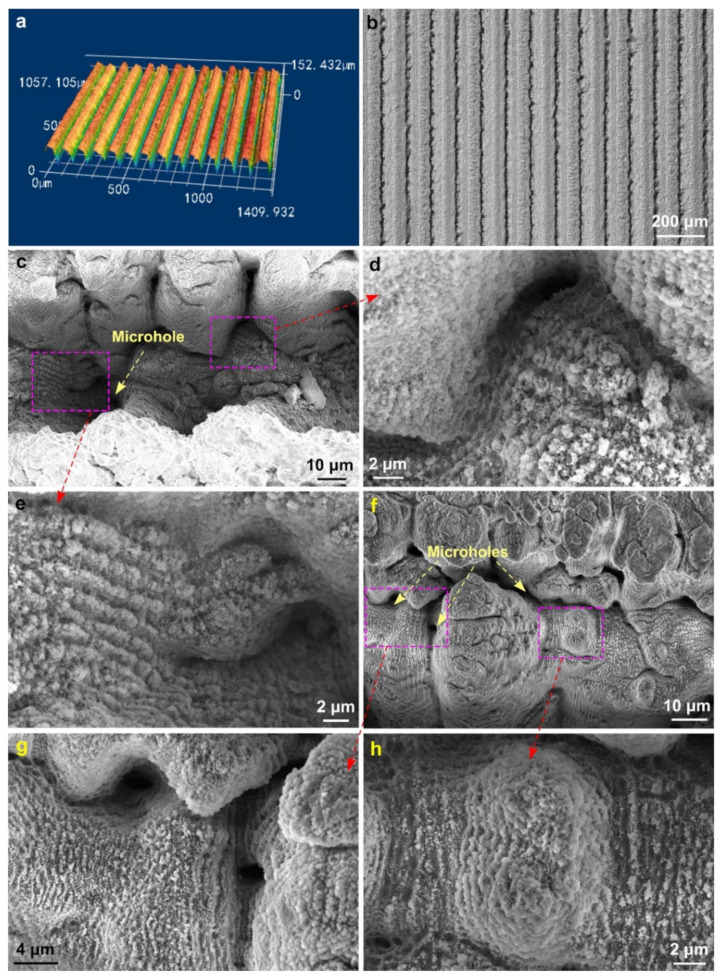
(**a**) Three-dimensional optical image of the array of parallel microgrooves. (**b**) Optical microscope image of the array of parallel microgrooves. (**c**) SEM image of a microgroove valley with areas textured by laser-induced periodic surface structures (LIPSS). (**d**) Magnified SEM image of LIPSS-textured area near microgroove wall. (**e**) Magnified SEM image of LIPSS-textured area in a microhole. (**f**) SEM image of a microgroove ridge with LIPSS-textured areas. (**g**) Magnified SEM image of LIPSS-textured area in ridge microholes. (**h**) Magnified SEM image of LIPSS-textured area around a ridge microcolumn.

The period and averaged depth of the basic capillary microgrooves are measured to be 100 ± 12 µm and 88 ± 7 µm, respectively. The surface textures produced on a microgroove bottom and ridge are shown in Figure 2. As seen, in addition to common random nano/microstructures, the microgroove texture includes a large number of LIPSS areas and fine microholes with a LIPSS-textured surface. The microhole formation results from a “keyhole” effect observed after ablation with continuous wave (CW), long-pulse, and ultrashort-pulse lasers [86,87,88,89,90]. This effect is caused by the formation of microspots with enhanced laser beam absorption at some surface structures on the crater bottom that results in a higher ablation rate and the creation of a microhole that acts as a focusing cavity and further enhances the laser beam absorption due to multiple reflections [86,87,88,89]. It has been previously found that the formation of random deep microholes at the crater bottom following static multipulse femtosecond laser ablation is observed at laser fluences above ~3 J/cm^2^ [88]. Under our dynamic scanning ablation conditions, the fine microholes form at *F* > 5.1 J/cm^2^. Both increasing the laser fluence and decreasing the scanning speed result in a larger number of produced microholes. The diameter and depth of the microholes in our capillary structure are mostly in a range of 5–30 µm and 5–40 µm, respectively. As seen in Figure 2, the LIPSS areas are randomly formed on the ridge, bottom, and walls of a microgroove. Due to the geometrical complexity of the microgroove structure, the LIPSS morphology significantly varies. An important factor in variation of the LIPSS morphology is the laser beam incidence angle that determines the LIPSS period. The LIPSS period *d* on a metal irradiated with a linearly polarized laser light in an ambient dielectric medium is given by Equation (1) [1,19,91,92]:

(1) d=λlas/Reη∓sinθi, with g∥E

where *λ_las_* is the wavelength of incident laser light, *θ_i_* is the angle of laser beam incidence, $\eta ={\left[\varepsilon_{d}\varepsilon_{metal}/(\varepsilon_{d}+\varepsilon_{metal})\right]}^{1/2}$ is the effective refractive index of the dielectric–metal interface for surface plasmons, *ε_d_* is the dielectric constant of the ambient dielectric medium, *ε_metal_* is the dielectric constant of the metal, Re[*η*] is the real part of *η*, **g** is the grating vector of LIPSS, and **E** is the tangential component of electrical field vector of the incident laser beam. Typically, the period of LIPSS generated by the femtosecond pulses at *θ_i_* ≈ 0 is in a range between 100 and 600 nm. Depending on the laser ablation conditions, larger LIPSS periods can be also produced at a normal incidence angle [93]. At *θ_i_* > 0, the LIPSS period increases, being in a range of 1–4 µm at 30° < *θ_i_* < 80° [91]. Another important factor in the variation of the LIPSS period is the effective refractive index of the dielectric–metal interface *η* for surface plasmons that depends on morphology of fine nanostructures formed during multipulse ablation [30]. The major laser parameters that govern the morphology of fine microstructures are laser fluence and number of overlapping laser pulses [1]. In our study, the number of overlapping laser pulses is 120. Figure 2 shows that the microgroove surface is textured with random both nano- and fine microstructures of various shapes typically formed by multipulse femtosecond laser ablation [1]. The dimensions of these random structures are in a range between 35 nm and 5 µm.

**Figure 3:**
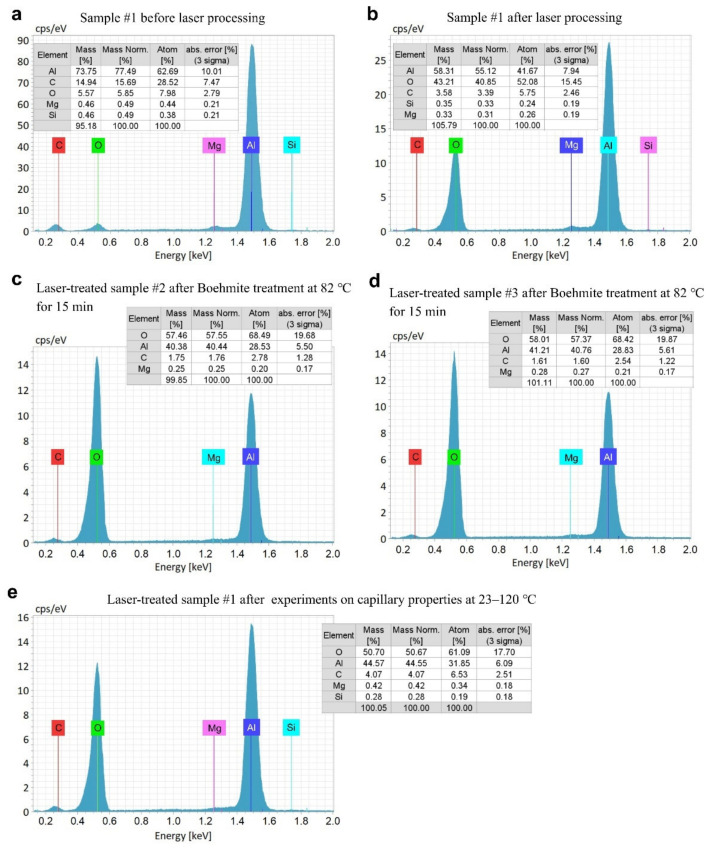
(**a**) Elemental composition of the sample #1 surface before laser processing. (**b**) Elemental composition of a sample surface after laser processing (sample #1). (**c**) Elemental composition of the sample surface after water treatment at 82 °C for 15 min (sample #2). (**d**) Elemental composition of the sample surface after water treatment at 100 °C for 15 min (sample #3). (**e**) Elemental composition of the sample #1 after water capillary flow experiments at 23–120 °C.

Previously, it has been shown that femtosecond laser ablation in air causes aluminum surface to oxidize [94]. The elemental compositions of an aluminum surface before and after laser processing are demonstrated in Figure 3a,b, respectively. One can see that oxygen content after laser processing increases by about seven times. The treatments of the laser-processed samples in hot water results in further increasing the oxygen content (see Figure 3c,d). Since the capillary flow of water on laser-treated aluminum without Boehmite treatment (sample #1) is studied at surface temperatures ranging from 23 to 120 °C, the elemental composition of the surface can be modified by chemical interaction of hot aluminum surface with water during experiments in a way similar to Boehmite treatment. To assess this modification, we performed an EDS analysis of the sample #1 after our experiments on the water spreading dynamics. The results of this EDS analysis, shown in Figure 3e, reveal the increase in oxygen content. This oxygen increase is slightly smaller than that on samples #2 and #3 after hot water treatments as can be seen from a comparison of Figure 3c–e. The laser-treated samples appear pitch black after laser processing, demonstrating black-metal optical properties with broadband light absorption [1]. However, all the studied samples exhibit some loss of their blackness after contact with hot water, indicating a degradation of light absorption properties caused by AlO(OH) surface layer formation.

**Figure 4:**
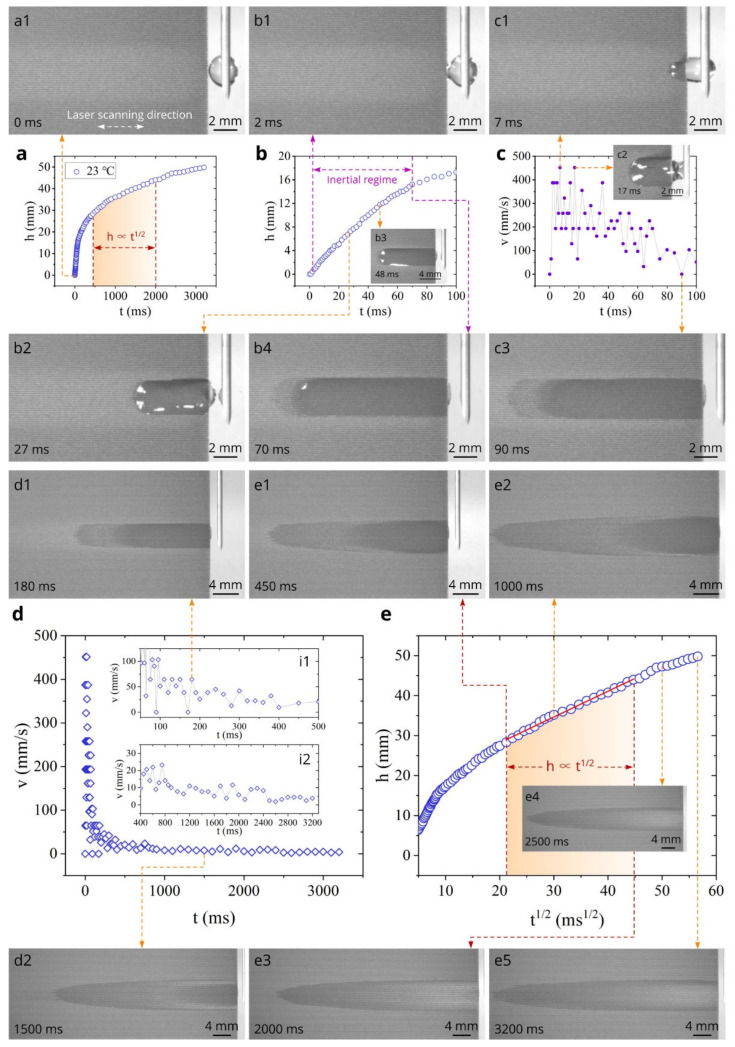
Experimental *h*(*t*) and *v*(*t*) plots along with snapshots of water spreading at 23 °C for sample #1. (**a**) Overall plot of the spreading distance as a function of time between 0 and 3200 ms (a1 is a snapshot of the sample surface and water drop at *t* = 0). (**b**) Detailed plot of the spreading distance as a function of time between 0 and 100 ms (b1–b4 are snapshots of water spreading associated with *h*(*t*) dependence). (**c**) Plot of the velocity as a function of time between 0 and 100 ms (c1–c3 are snapshots of water spreading associated with *v*(*t*) dependence). (**d**) Overall plot of the velocity as a function of time between 0 and 3200 ms (d1, d2 are snapshots of water spreading associated with *v*(*t*) dependence. The insets i1 and i2 show detailed *v*(*t*) dependencies in the time domains of 100–500 and 400–3200 ms, respectively). (**e**) Plot of the spreading distance as a function of *t*^1^^/^^2^ (e1–e5 are snapshots of water spreading associated with Washburn’s regime). (Note: Bleaching of surface in the middle of the sample seen in Snapshots of both this and Figure 5 and Figure 6 is discussed in paper later).

The data on the capillary flow dynamics on the surface of sample #1 at room temperature (*T* = 23 °C) are presented in Figure 4. The plot of the water spreading distance *h* as a function of time *t* is shown in Figure 4a. It is seen that water spreads for a distance of 50 mm in about 3000 ms, demonstrating a very fast capillary flow. Figure 4b shows *h*(*t*) dependence in the initial time domain 0 < *t* < 100 ms, where acceleration and inertial stages of the capillary flow typically take place [64,85,95,96,97,98,99]. It is seen that the spreading distance achieves an extremely large value of 17.3 mm at 100 ms. The plot of the capillary flow velocity *v* as function of time in Figure 4c demonstrates an extraordinarily high spreading velocity that reaches a maximum value of 452 mm/s in the initial capillary flow stage. This value of the maximum spreading velocity exceeds those that have been previously observed in 1D microgroove structures (370 mm/s in [85] and 120 mm/s in [100]). Using the same approach for identifying the inertial regime as in [64,85], we find in our study that the inertial regime, where the spreading distance is a quasilinear function of time, lasts until about 70 ms. Snapshots a1, b1, b2, b3, b4, c1, c2, and c3 illustrate the water spreading in the time domain 0 < *t* < 90 ms. The physical processes of the studied here capillary flow are similar to those in the 1D array of microgrooves on silicon reported in [85], where a detailed discussion of the mechanisms governing water flow is given. Figure 4d shows the overall *v*(*t*) plot in the time domain 0 < *t* < 3200 ms. It is seen that the velocity quickly decreases after the inertial flow stage down to about 15–20 mm/s at *t* = 400 ms (see insets i1 and i2). This quick decrease of the velocity occurs in the visco-inertial capillary flow regime that takes place between the inertial (*h* ∝ *t*) and Washburn (*h* ∝ *t*^1/2^) regimes [101,102]. The liquid flow dynamics in the visco-inertial regime follows the relationship *h* ∝ *t*^n^, where *n* gradually reduces from 1 to 0.5 [102]. Snapshots c3 and d1 illustrate the water flow in the visco-inertial stage. To identify the time domain of Washburn’s *t*^1/2^ regime, we plotted *h* as a function of *t*^1/2^ in Figure 4e, where one can see that the Washburn capillary flow takes place between 400 and 2000 ms. Snapshots e1, e2, and e3 show the water flow in the Washburn regime. As seen in Figure 4d and its insets i1 and i2, the decrease in the spreading velocity is slow during both Washburn’s stage and the final flow stages as compared with that in the visco-inertial regime. The water spreading in the final capillary flow stages is demonstrated by Snapshots e4 and e5.

**Figure 5:**
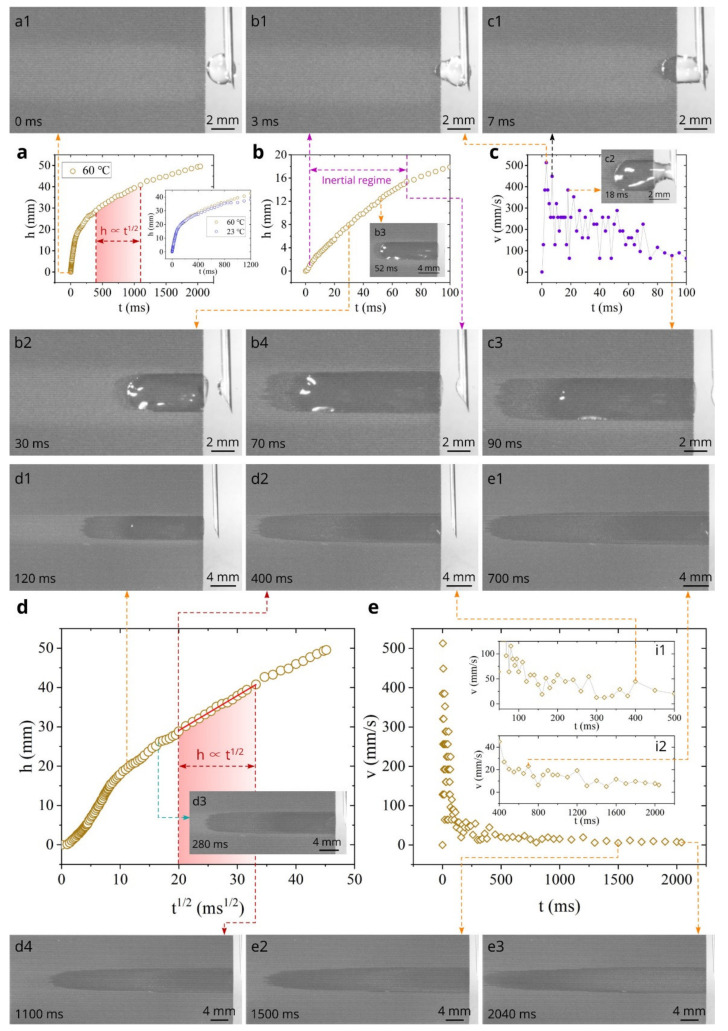
Obtained *h*(*t*) and *v*(*t*) plots along with snapshots of water spreading at 60 °C for sample #1. (**a**) Overall plot of the spreading distance as a function of time between 0 and 2040 ms (a1 is a snapshot of the sample surface and water drop at *t* = 0). (**b**) Detailed plot of the spreading distance as a function of time between 0 and 100 ms (b1–b4 are snapshots of water spreading associated with *h*(*t*) dependence). (**c**) Plot of the velocity as a function of time between 0 and 100 ms (c1–c3 are snapshots of water spreading associated with *v*(*t*) dependence). (**d**) Plot of the spreading distance as a function of *t*^1^^/^^2^ (d1, d3 are snapshots of water spreading related to visco-inertial regime; d2, d4 are snapshots of water spreading related to Washburn’s regime). (**e**) Overall plot of the velocity as a function of time between 0 and 2040 ms (e1–e3 are snapshots of water spreading associated with *v*(*t*) dependence. The insets i1 and i2 show detailed *v*(*t*) dependencies in the time domains of 100–500 and 400–2040 ms, respectively).

Figure 5 presents the data on the capillary flow dynamics of sample #1 at 60 °C. The plot of the spreading distance as a function of time in Figure 5a shows that the water spreading distance of 50 mm is achieved in 2040 ms, demonstrating a faster capillary flow than that at 23 °C. The inset in Figure 5a demonstrates a comparison of the *h*(*t*) dependences at 23 and 60 °C. It is seen that at 0 < *t* < 400 ms the spreading distance is about the same, indicating a negligible temperature effect on water flow. However, the temperature effect on the water spreading clearly manifests itself at *t* > 400 ms. The capillary action enhancement with increasing temperature has been previously explained in [85]. The detailed *h*(*t*) dependence at 0 < *t* < 100 ms in Figure 5b shows a very large spreading distance in the initial stage, reaching about 17.9 mm at *t* = 100 ms. The inertial flow regime, where *h* is a quasilinear function of time [64,85], occurs between 3 ms and about 70 ms, as indicated in Figure 5b. The *v*(*t*) plot in Figure 5c reveals an acceleration stage between 0 and 3 ms, where the capillary flow velocity increases from 0 to an extremely high value of about 513 mm/s. Water spreading in the end of this acceleration stage is illustrated by Snapshot b1. The initial water spreading dynamics in the time domain 0 < *t* < 90 ms are demonstrated by Snapshots a1, b1, c1, c2, b2, b3, b4, and c3. Water spreading in the inertial regime is illustrated by Snapshots b1, c2, b2, b3, and b4. The plot *h*(*t*^1/2^) in Figure 5d shows that the Washburn regime occurs between 400 and 1100 ms, as indicated in Figure 5a. Thus, the visco-inertial regime, where *h* ∝ *t*^n^ with 1 > *n* > 0.5, takes place between 70 and 400 ms. Figure 5e presents the overall *v*(*t*) dependence in the entire studied time domain (0 < *t* < 2040 ms). It is seen that the velocity significantly reduces in the visco-inertial regime down to about 25 mm/s (see Figure 5e and its inset i1). Snapshots c3, d1, d2, and d3 demonstrate water spreading in the visco-inertial regime. In the Washburn stage (400 < *t* < 1100 ms), the velocity decreases to about 12 mm/s, as seen in Figure 5e and its inset i2. Snapshots d2, e1, and d4 illustrate the water spreading in the Washburn stage. Water spreading after Washburn’s stage is demonstrated in Snapshots e2 and e3. In summary, the data obtained in the temperature range between 23 and 60 °C show the excellent wicking properties of the created aluminum material, indicating its practical applicability in M-cycle air-conditioners for enhancing their efficiency [76,79,103,104].

**Figure 6:**
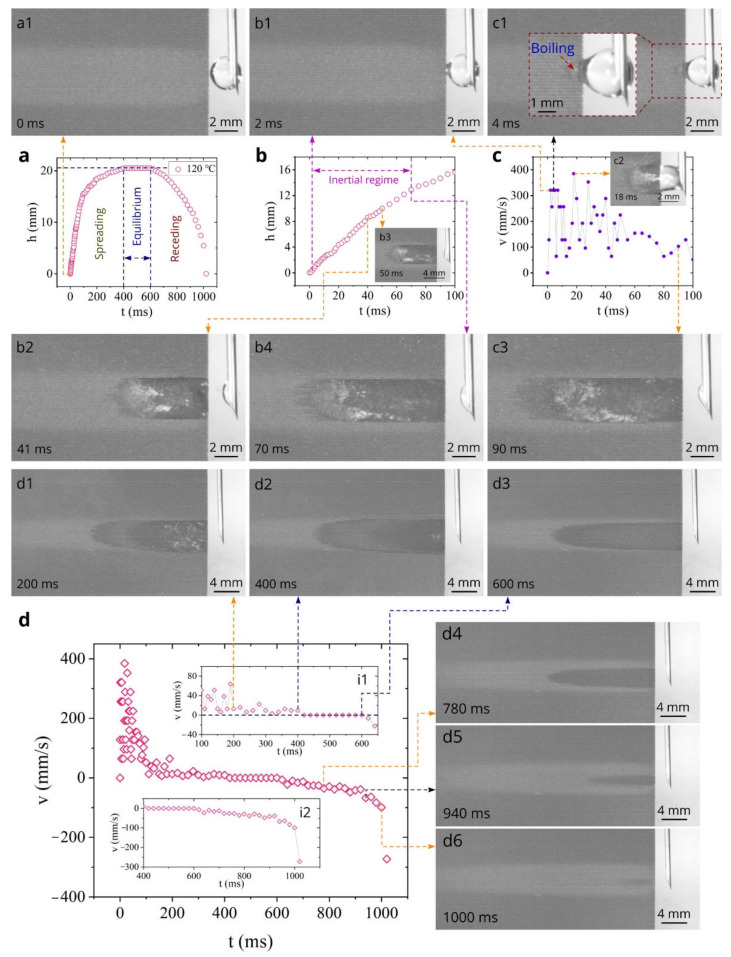
The *h*(*t*) and *v*(*t*) dependencies at 120 °C and snapshots of water behaviors in spreading, equilibrium, and receding regimes for sample #1. (**a**) The overall plot of the spreading distance as a function of time (a1 is a snapshot of the sample surface and water drop at *t* = 0). (**b**) Detailed *h*(*t*) plot in the time domain between 0 and 100 ms (b1–b4 are snapshots of water spreading associated with *h*(*t*) dependence). (**c**) Detailed plot of the spreading velocity as a function of time between 0 and 100 ms (c1–c3 are snapshots of water spreading associated with *v*(*t*) dependence). (**d**) The overall plot of spreading and receding velocities as a function of time (d1–d6 are snapshots of water spreading associated with overall *v*(*t*) dependence. The insets i1 and i2 show detailed *v*(*t*) dependencies in the time domains of 100–650 and 400–1020 ms, respectively).

The wicking dynamics of laser-fabricated materials have been previously studied at temperatures below the boiling point of water. Here, we extend the studied temperature range up to 120 °C. Figure 6 presents our results on the water spreading, boiling, and receding (drying) dynamics obtained at 120 °C. The overall *h*(*t*) dependence is demonstrated in Figure 6a, where it is seen that initially the water front spreads on the hot surface, reaches a maximum spreading distance of 20.5 mm at *t* = 400 ms, remains immovable at 400 < *t* < 600 ms, and then recedes due to evaporation until the complete evaporation of water at *t* = 900 ms. These water front behavior stages are indicated in Figure 6a as “spreading”, “equilibrium”, and “receding”. Snapshots d2 and d3 show that although the spreading distance remains about the same in the equilibrium stage, the wetted surface area becomes narrower with time. The detailed *h*(*t*) and *v*(*t*) plots in the time domain 0 < *t* < 100 ms shown in Figure 6b,c demonstrate that the water spreading distance achieves about 15.6 mm at 100 ms and the maximum spreading velocity attains about 320–325 mm/s between 2 and 4 ms, exhibiting an extremely strong capillary action even at the temperature above the boiling point of water. Snapshots a1, b1, b2, b3, c1, c2, and c3 illustrate the water behaviors in the time domain 0 < *t* < 100 ms. Snapshot b1 shows the water spreading in the end of the acceleration stage (*t* = 2 ms) when the spreading velocity achieves about 325 mm/s. The higher velocities of about 385 mm/s and 353 mm/s are observed at *t* = 18 and 28 ms, respectively. These velocity enhancements are correlated with big bubble bursts that can push water forward. As seen in Snapshots c1, boiling begins at *t* = 4 ms and completes at about 400 ms (see Snapshot d2) when the equilibrium stage is almost finished. The evolution of boiling in the spreading water film is illustrated by Snapshots c1, c2, b2, b3, b4, c3, d1, and d2. It is seen that, initially, the boiling area closely follows the water spreading front (see Snapshots c1, b2, c2, and b4). At a later time, the boiling area spreading stops and it begins receding towards to the right edge of the sample. As seen in Snapshot d2, boiling terminates near the right edge of the sample at about 400 ms when the spreading stage comes to the end. This evolution of the boiling area is explained by the decrease in the water film thickness to a level of 1–10 µm that does not support nucleate boiling [105]. After the boiling termination, the water film thickness becomes small in the entire wetted area, thus causing evaporation to occur through a thin film evaporation mechanism [106] that governs the water behavior in the equilibrium and receding stages. The overall plot of the spreading and receding velocities as functions of time is shown in Figure 6d. The inset i1 shows in detail the *v*(*t*) dependence in the end of spreading stage and in the equilibrium stage, where *v* ≈ 0 mm/s. The dynamics of the evaporating water film in the receding regime is illustrated by Snapshots d3 to d6. It is seen that after the equilibrium stage, the water film front begins to recede with an increasing velocity, achieving an extremely high value of about 272 mm/s in the end of the receding stage (see the inset i2). As seen in Snapshot d6, the sample surface becomes completely dry at about 1020 ms. Washburn’s dynamics of water spreading was not found at 120 °C. In summary, our study on wicking dynamics at 120 °C shows the excellent wicking performance of the developed material even above the boiling point of water.

**Figure 7:**
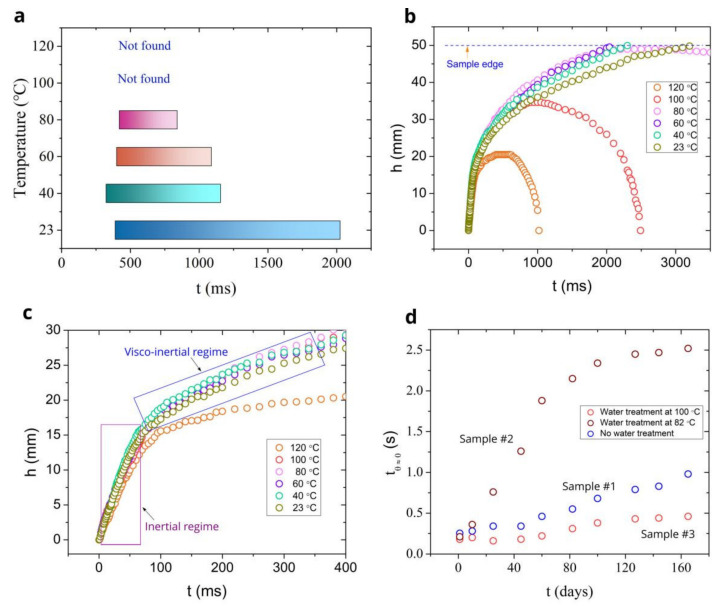
(**a**) Diagram of Washburn’s regime lifetime at different temperatures and air pressure of 1 atmosphere. (**b**) Comparison of *h*(*t*) dependences at different temperatures in the entire studied time domain. (**c**) Comparison of *h*(*t*) dependences at different temperatures in the time domain 0 < *t* < 400 ms. (**d**) The *t*_θ≈0_ (*t*) plots of the studied samples.

The classic Washburn *h* ∝ *t*^1^^/^^2^ flow dynamics [107] is observed in many capillary systems [12,95,100,108,109,110,111,112,113]. It is often considered as a universal one, being the standard in comparing capillary flows [114]. For smooth V-microgrooves, Washburn’s capillary flow is given by *h*^2^ = *K*(*α,θ*)[*γd/μ*]*t*, where *K*(*α,θ*) is the geometry term with *α* being the groove angle, *θ* is the contact angle, *d* is the groove depth, *γ* is the liquid surface tension, and *µ* is the liquid viscosity [109]. In the Washburn regime, the flow of a liquid at room temperature is governed by capillary and viscous forces [107]. At elevated temperatures, it can be affected by evaporation. Previously, it has been found that the *h* ∝ *t*^1^^/^^2^ regime lifetime decreases with increasing temperature due to effect of evaporation that deviates the liquid flow from the Washburn dynamics [85]. Figure 7a presents our data on the lifetime of Washburn’s regime in the studied temperature range at air pressure of 1 atmosphere. It is seen that the Washburn regime is observed in the temperature range between 23 and 80 °C. Its onset time remains almost unchanged but its termination time significantly shortens as the temperature increases. The Washburn dynamic is not observed above *T* ≥ 100 °C, indicating a significant role of evaporation/boiling. To understand the evaporation and boiling effects on the liquid flow dynamics, we perform a comparative analysis of the *h*(*t)* dependencies obtained at the studied temperatures. A comparison of the *h*(*t*) dependencies at different temperatures in the entire studied time domain is presented in Figure 7b. It is seen that all *h(t*) dependences are about the same in the time domain between 0 and 100 ms, where the inertial and partially visco-inertial flow regimes occur. At a later time, one can clearly see the effect of the spreading distance increase as the temperature rises between 23 and 80 °C. A detailed comparison of the *h(t*) dependencies for demonstration of the temperature effect on water spreading in the inertial and visco-inertial flow regimes at 0 < *t* < 400 ms is shown in Figure 7c. This water spreading behavior can be explained by using equations of capillary flow in the inertial [101], visco-inertial [101], and Washburn [107,109] regimes, which show that, in general, the spreading of a liquid can be affected by such temperature-dependent parameters as the contact angle, surface tension, viscosity, and density *ρ* of the liquid. The water contact angle on an aluminum surface is relatively constant for surface temperatures ranging from 25 to 120 °C [115]. In the context of capillary flow, the temperature dependences of *γ*, *μ*, and *ρ* for water have been previously discussed in [85] and are shown to be favorable for enhancing capillary action with increasing temperature. Therefore, the capillary flow enhancement in our work comes from the temperature dependences of *γ*, *μ*, and *ρ*. The small temperature effect on water spreading in the inertial regime is explained by the fact that a certain amount of time is needed for water heating. This time can be estimated from the equation for thermal diffusion length *L* given by *L* ≈ (*Dt*)^1/2^, where *D* is the thermal diffusivity of a material. Assuming that the water film thickness in our capillary structure is about the depth of the microgrooves (88 µm), we can estimate a characteristic timescale *t*_d_ of heating the water in a microgroove by taking *L* = 88 µm and using a table value of *D* = 0.0014 cm^2^ s^−1^ for water. This estimation gives us *t*_d_ ≈ *L^2^/D* ≈ 55 ms, which is in an agreement with *t* ≈ 40–60 ms in Figure 7c when temperature effect on water spreading begins to play a role. It is seen in Figure 7c that *h(t*) dependences are almost unaffected by evaporation in the inertial and visco-inertial regimes at 23 ≤ *T* ≤ 100 °C. At *T* = 120 °C, water spreading in the inertial regime (*t* < 70 ms) is slightly affected by boiling; however, the boiling effect on water spreading becomes significant at *t* > 80 ms.

The common adsorption of hydrophobic hydrocarbons from the ambient air onto metal surfaces often results in the quick degradation of their hydrophilic/capillary properties [21,116,117], making the creation of long-term stable wicking materials a challenging task. It is known that the treatment of aluminum in hot water improves its both hydrophilic/wicking properties and long-term stability [80,81,82,83]. These effects result from both the modification of the surface chemistry and the formation of “grass-like” surface nanostructures [82]. Our study on wicking dynamics performed during one week after samples fabrication shows that the wicking properties of the samples treated in hot water (samples #2 and #3) are about the same as those of the sample #1. This observation indicates that femtosecond laser processing provides excellent hydrophilic/wicking properties, making their further improvement with Boehmite treatment a difficult task. To assess the long-term wicking stability of our samples, we use video recording to obtain the time dependence of the dynamic contact angle *θ* of a water drop immediately after its deposition onto the sample that allows to find the time *t*_θ≈0_ when *θ* decreases to static zero value (*θ* ≈ 0°) [64]. To perform these measurements, we use an OSA 200 system for measuring the contact angle in video recording mode at a speed of 50 fps. The results obtained over a test period of 170 days are presented in Figure 7d as the *t*_θ≈0_ (*t*) plots. These plots show that the contact angle becomes zero at about the same *t*_θ≈0_ ≈ 0.25 s for all three studied samples tested during few days after their fabrication. However, after 170 days, the time *t*_θ≈0_ becomes 1.0, 2.6, and 0.48 s for the samples #1, #2, and #3, respectively. These data show that sample #2 treated in water at 82 °C exhibits faster wicking degradation with time as compared with sample #1, while sample #3 treated in water at 100 °C demonstrates a slower degradation. Thus, our study shows that the hot water treatment at 100 °C can improve the long-term wicking stability of aluminum materials produced by femtosecond laser processing. This finding is important for developing practical long-term stable wicking Al materials.

**Figure 8:**
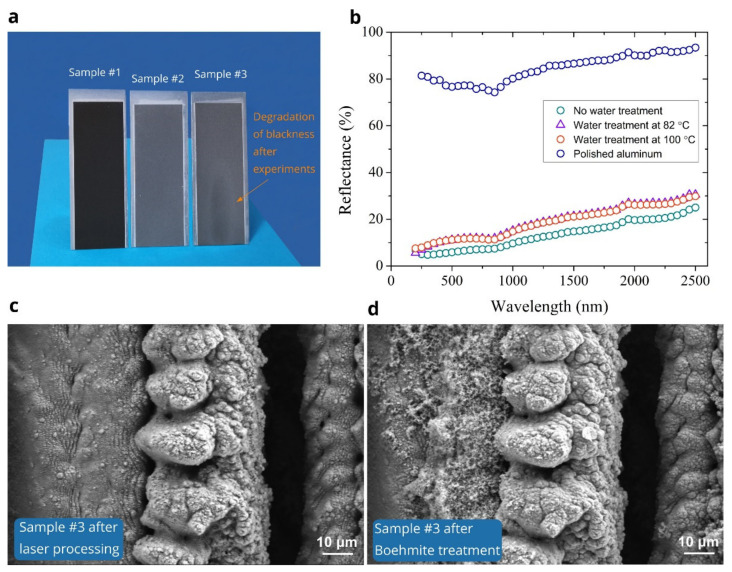
(**a**) Photos of the samples #1 (after laser processing), #2 (after Boehmite treatment at 82 °C), and #3 (Boehmite-treated at 100 °C and studied for wicking properties). (**b**) Reflectance of aluminum samples as a function of the light wavelength *λ*. For a comparison, the *R*(*λ*) of a polished sample before laser processing is also shown. (**c**) SEM image of the surface of the sample #3 after laser processing. (**d**) SEM image of the same surface of the sample #3 after Boehmite treatment.

The femtosecond laser processing that we use also renders the optical properties of a black metal [1] to our samples, in addition to their wicking functionality. Due to high optical absorption, the laser-treated surface appears pitch black (see photo of the sample #1 in Figure 8a). However, we find that the surface blackness degrades after Boehmite hot water treatments as seen in photos of the samples #2 and #3 in Figure 8a. The blackness degradation is also observed in our samples after experiments on water spreading at elevated temperatures (see the photo of sample #3 in Figure 8b). To assess the morphological changes caused by Boehmite treatment, we took SEM images of sample #3 after both laser processing and Boehmite treatment shown in Figure 8c,d, respectively. It is seen that the Boehmite treatment causes both fine micro- and nanostructural modifications. Highly absorptive optical properties of black metals stem dominantly from the plasmonic absorption of light by surface metal nanostructures [1]. Therefore, the observed blackness degradation can be explained by the depletion of aluminum nanostructures caused by their oxidation during the Boehmite treatment. This also explains the surface bleaching of sample #1 seen in Snapshots in Figure 4, Figure 5 and Figure 6 as a consequence of significant oxidation of the sample #1 surface during our experiments as evidenced by the EDS analysis shown in Figure 3e. To quantitatively characterize the visually observed change of the optical properties, we measure the total (specular + diffuse) reflectance *R* of our samples in a wavelength range of 250–2500 nm using a Shimadzu spectrophotometer UV-3600 equipped with an integrating sphere. The obtained *R(λ)* dependencies are demonstrated in Figure 8b. For a comparison, the *R(λ)* of a polished sample before laser processing is also shown. These data demonstrate that the interaction of aluminum with hot water reduces the optical absorptance of the laser-treated aluminum materials, thus limiting their multifunctional applications in technological areas where both wicking and solar energy harvesting properties are needed.

## 4. Conclusions

In summary, we produced a hierarchical capillary microgroove surface structure that includes both LIPSS and fine microhole textures using the direct femtosecond laser nano/microstructuring of aluminum. The developed material was tested for wicking performance in a wide temperature range of 23–120 °C at an air pressure of 1 atmosphere. The tests show an extremely high velocity of water spreading that achieves about 450 mm/s at 23 °C and 320 mm/s at 120 °C when the spreading water undergoes intensive boiling. The analysis of the dynamic water behavior demonstrates the decrease of the Washburn regime lifetime with increasing temperature up to 80 °C. The effects of evaporation and boiling on water spreading become significant at *T* > 80 °C, resulting in vanishing of Washburn’s dynamics. At temperatures below the boiling point, evaporation insignificantly affects both the inertial and visco-inertial flow regimes. A pronounced effect of boiling on the visco-inertial regime is found at 120 °C; however, the boiling effect on the inertial regime is small. We find that the hot water treatment at 100 °C improves the long-term wicking stability of laser-fabricated wicking aluminum materials. The strong capillary action of the created material under water boiling conditions extends the range of its potential applications at high temperatures, such as boilers with enhanced critical heat flux [82,118,119] and M-cycle heat/mass exchangers for enhancing energy efficiency in power generation [76,77,78,79,120,121,122].

## Data Availability

Data are contained within the article.
